# Ontogenetic stage-specific reciprocal intraguild predation

**DOI:** 10.1007/s00442-018-4256-6

**Published:** 2018-09-01

**Authors:** Morgana Maria Fonseca, Angelo Pallini, Eraldo Lima, Arne Janssen

**Affiliations:** 1 Department of Entomology, Federal University of Viçosa, Viçosa, Minas Gerais Brazil; 2 Evolutionary and Population Biology, IBED, University of Amsterdam, Science Park 904, 1098 XH Amsterdam, The Netherlands

**Keywords:** Predator–predator interactions, Stage structure, Ontogenetic diet shifts, Predatory mites, Biological control

## Abstract

The size or stage of interacting individuals is known to affect the outcome of ecological interactions and can have important consequences for population dynamics. This is also true for intraguild predation (the killing and eating of potential competitors), where the size or ontogenetic stage of an individual determines whether it is the intraguild predator or the intraguild prey. Studying size- or stage-specific interactions is therefore important, but can be challenging in species with complex life histories. Here, we investigated predatory interactions of all feeding stages of the two predatory mite species *Neoseiulus californicus* and *Phytoseiulus macropilis*, both of which have complex life cycles, typical for predatory arthropods. Populations of these two species compete for two-spotted spider mites, their prey. We evaluated both the capacity to kill stages of the other predator species and the capacity to benefit from feeding on these stages, both prerequisites for the occurrence of intraguild predation. Ontogeny played a critical role in the occurrence of intraguild predation. Whereas the juveniles of *P. macropilis* developed from larva until adulthood when feeding on *N. californicus* eggs, interestingly, adult female *P. macropilis* did not feed on the smaller stages of the other species. We furthermore show that intraguild predation was reciprocal: both juveniles and adult females of *N. californicus* preyed on the smallest stages of *P. macropilis*. These results suggest that a proper analysis of the interactions between pairs of species involved in intraguild predation should start with an inventory of the interactions among all ontogenetic stages of these species.

## Introduction

The type and strength of ecological interactions frequently depend on the the size or stage of the interacting individuals (Werner and Gilliam 1984; Polis and Strong 1996; Rudolf and Lafferty 2011). Because predators often begin their lives small and vulnerable, this has consequences for their ecological role, for example, they may compete for resources with individuals of another species when small and prey on the same species when large (Wilbur 1988). Moreover, an individual acting as a predator when adult and large may be vulnerable to attacks and be killed by large adult prey when small (Polis et al. 1989; Polis 1991; Palomares and Caro 1999; Janssen et al. 2002) and this can result in complex dynamics (Persson et al. 2007a; Van Leeuwen et al. 2008; de Roos and Persson 2013). Such killing of small predators may arise both within species through cannibalism (Elgar and Crespi 1992) and among species through hyperpredation (Rosenheim 1998), intraguild predation (Polis et al. 1989; Polis 1991; Palomares and Caro 1999), omnivory (Faraji et al. 2001; Janssen et al. 2002, 2003) and, intriguingly, even through predation by prey that are considered purely herbivorous (Aoki et al. 1984; Saito 1986).

Intraguild predation (IGP), the killing and eating of heterospecific competitors, is a widespread interaction (Polis et al. 1989; Arim and Marquet 2004) and has important consequences for population dynamics, species coexistence and community structure (Mylius et al. 2001; Woodward and Hildrew 2002; Persson et al. 2007b; Schröder et al. 2009; Montserrat et al. 2012; de Roos and Persson 2013; Preston et al. 2014; Toscano et al. 2016, 2017). Often, the stage or size of the individual determines whether it is an intraguild predator or intraguild prey. This is frequently studied or modelled as large, adult individuals of one species feeding on juveniles of the other species (Polis et al. 1989; Mylius et al. 2001; Woodward and Hildrew 2002; Montserrat et al. 2012). Although this is a good first approximation, interactions among species with complex life cycles may require a further subdivision into interactions between the ontogenetic stages of the involved individuals. For example, first-instar larvae of the thrips species *Frankliniella occidentalis* are vulnerable to predation by older stages and adults of several species of predatory mites, but second-instar larvae and adults are invulnerable (Bakker and Sabelis 1989; Belliure et al. 2008). Prepupae, the stage between second-instar larvae and pupae, have the size of a full-grown second-instar larva, but are again vulnerable to attacks by the predatory mites (K. Muñoz-Cárdenas and M. Duarte, pers. obs.). In return, both first- and second-instar and adult thrips prey on eggs of the predatory mites (Janssen et al. 2002; de Almeida and Janssen 2013). This shows that both size and stage determine predation risk.

In stage-structured systems, different stages of interacting predatory species often coexist, and two co-occurring predator species may attack each other’s vulnerable stages (Polis 1984; Choh et al. 2012; Montserrat et al. 2012; Marques et al. 2018), thus engaging in a more complex type of IGP, termed reciprocal intraguild predation (RIGP), which is also observed in natural systems (Polis et al. 1989; Wissinger 1992; Woodward and Hildrew 2002; Marques et al. 2018). Because of the importance of intraguild predation and reciprocal intraguild predation for population dynamics and species coexistence, it is necessary to investigate the interactions among all stages of species pairs that are suspected to be involved in these interactions. Here, we quantified predation of two predator species by feeding stages of the other species.

Some of the best studied systems with intraguild interactions consist of biological control systems, especially in greenhouse crops, because these artificial food webs (Ehler 1996) are easier to manipulate than natural systems (Messelink et al. 2012). The increased use of biological control agents in agricultural crops has led to a growing complexity of these artificial food webs; hence, the densities of the target pests species are increasingly determined by the joint effects of various direct and indirect interactions among prey and predators (Janssen et al. 1998; Rosenheim et al. 1999; Cakmak et al. 2006; Messelink et al. 2012; van Lenteren et al. 2018). With the presence of several natural enemies, particularly generalist predators, IGP can occur more frequently and is increasingly considered in the development of successful biological control programmes (Rosenheim et al. 1993, 1995, Janssen et al. 1998, 2006; Messelink and Janssen 2014; Wells et al. 2017). Here, we investigated the interplay between ontogenetic stages and IGP interactions in a system consisting of two biological control agents: the predatory mite species *Neoseiulus californicus* (McGregor) and *Phytoseiulus macropilis* (Banks), (Acari: Phytoseiidae). Our aim was to evaluate whether these predatory mites interact through intraguild predation and, if so, which stages are involved in this.

## Materials and methods

### The experimental system

The two predatory mites are mass produced in several regions of the world and used as biological control agents of the two-spotted spider mite *Tetranychus urticae* [Koch (Acari: Tetranychidae)] (McMurtry and Croft 1997; Gerson et al. 2003), which is a cosmopolitan pest of over 1100 plant species and is resistant to many pesticides (Van Leeuwen et al. 2010; Migeon and Dorkeld 2015). In Brazil, both *P. macropilis* and *N. californicus* co-occur naturally in extensive regions on various plant species (Ferla et al. 2007; Roggia et al. 2009). *Phytoseiulus macropilis* is considered a specialist predator of *Tetranychus* species and tends to disperse from crops when the densities of prey are low (McMurtry and Croft 1997; Oliveira et al. 2007), whereas *N. californicus* has more generalist feeding habits and can thus feed on other food types in periods of low prey densities (Croft et al. 1998; Gerson et al. 2003). Therefore, these predators have been considered for combined releases to control *T. urticae*.

Both predators have five developmental stages: egg, larva, protonymph, deutonymph (the latter three here together referred to as juvenile) and adult. The size of each stage is similar for both species, and the duration of their development from egg to adult is about 5 days for both species (Escudero and Ferragut 2005; Souza-Pimentel et al. 2017). Both protonymphs and deutonymphs need to feed to complete their development and although most predatory mite larvae do not need to feed to reach the next stage, the larvae of our system were observed feeding. We systematically explored intraguild predation by juveniles and adults of the two species and verified whether reciprocal intraguild predation occurred. We evaluated both the capacity to kill stages of the other species as well as the capacity to benefit from feeding on these stages, both of which are prerequisites for the occurrence of IGP (Polis et al. 1989; Fonseca et al. 2017).

### Cultures

The two-spotted spider mite (*T. urticae*) was reared on jack bean plants [*Canavalia ensiformis* (L.) DC] in a climate-controlled room (25 ± 3 °C, 70–90% relative humidity, with controlled photoperiod 12:12 L:D). Clean jack bean plants were grown in a greenhouse until they were 2 weeks old and were subsequently added to the spider mite culture twice per week. The predatory mites *P. macropilis* and *N. californicus* were reared under the same conditions as above on detached bean leaves infested with two-spotted spider mites. These leaves were put in a plastic tray (*l* × *w* × *h* = 45 × 30 × 8 cm) that was placed inside a second, water-containing tray (55 × 40 × 10 cm) to prevent the mites from escaping. New bean leaves with spider mites were added to the cultures two to three times per week. The cultures of spider mites and predatory mites were started with individuals obtained from cultures from Econtrole Pesquisa & Consultoria Ltda (Viçosa, MG, Brazil). Both predatory mite species had been reared for about a year on jack bean leaves with two-spotted spider mites prior to the experiments.

### Experimental setup

The experimental units used for all experiments consisted of plastic Petri dishes (diameter 6 cm, 1.5 cm high). Each Petri dish contained a small piece of wet cotton wool as a water source and was closed with cling film (Alpfilm^®^, Alpfilm Indústria e Comércio de Plásticos Ltda, São Paulo, Brazil). All adults used in the experiments were gravid females, aged between 10 and 15 days since the egg stage. These adult females were placed singly in the experimental units and were starved for 24 h prior to the experiments to prevent possible effects from the previous diet. Only females that had oviposited during this starvation period were used.

Cohorts of newly laid eggs and larvae were obtained by transferring females from the cultures to separate arenas of bean leaves with spider mites and allowing them to lay eggs for 24 h. Subsequently, the eggs were separated on a new arena, and were checked for hatched larvae every 12 h. Preliminary experiments were done to check how many individuals to offer to avoid prey depletion. All experiments were conducted in a climate-controlled room (conditions as above).

### IGP of adult females on heterospecific eggs, larvae and adult females

We first evaluated the predation rate of adult females of both species on heterospecific eggs and larvae and the effect of this feeding on their oviposition rate to verify whether adult females gained from this predation (Polis et al. 1989; Fonseca et al. 2017). A single gravid female was placed in each experimental unit together with eight heterospecific eggs or larvae. To measure natural mortality, only eggs or larvae were placed in another set of arenas. One day later, the deflated eggs or the shrivelled larvae were counted as evidence of predation. All treatments were replicated 14 times for *N. californicus* with eggs and 18 times with larvae of the other species and for *P. macropilis* 28 times with eggs and 27 times with larvae of *N. californicus*.

The oviposition rates of adult females feeding on heterospecific eggs or larvae were measured by offering eight eggs or eight larvae to adult females of the other species. As controls, the oviposition rate of adult females without food was assessed on another set of arenas. Newly laid eggs were counted every 24 h and the adult females were transferred to new experimental units. The experiments lasted 5 days and were replicated 14 times for *N. californicus* feeding on eggs of *P. macropilis*, and lasted 4 days and was replicated 10 times for *N. californicus* feeding on larvae of *P. macropilis*. We did not measure oviposition rates for adult females of *P. macropilis* when feeding on eggs or larvae of the other species because the predation experiment showed no evidence of predation.

In another experiment, we investigated whether adult females ate adult females of the other species. One adult female of each species was placed in each experimental unit together with a heterospecific adult female. Individuals of both species were held separately in controls to measure natural mortality. The predation was assessed after 24 h and the number of replicates was 16 per treatment. This experiment showed no evidence of predation between the adult females and oviposition rates were therefore not measured.

### IGP of juveniles on heterospecific eggs and juveniles

To examine whether other ontogenetic stages were potential intraguild predators, we assessed the predation rates of juveniles of both species on heterospecific eggs and juveniles. To verify whether this predation resulted in a benefit for the predator, we furthermore assessed the development and survival of the juveniles when feeding on heterospecific eggs and juveniles. The first experiment was started by placing a newly hatched larva in each experimental unit together with six heterospecific eggs. To measure natural mortality of eggs and as control for juvenile survival, there were two further treatments: only eggs or only larvae without eggs. Every 24 h, the state (dead or alive) and developmental stage of the juveniles were checked and preyed eggs were counted. Subsequently, the juveniles were transferred to new experimental units with new eggs (without eggs in the treatment with only larvae), and the eggs that were incubated without larvae to assess natural egg mortality were also renewed. The experiments lasted until the juveniles reached adulthood or died. Each treatment was replicated 12 times for *N. californicus* juveniles and 16 times for *P. macropilis* juveniles.

In another experiment, we investigated whether juveniles ate juveniles of the other species. One newly hatched larva of each species was placed in each experimental unit together with a newly hatched heterospecific larva. Individuals of both species were held separately in controls. The predation and developmental stage of juveniles were assessed every 24 h and the number of replicates was 14 per treatment. This experiment lasted 2 days, when predators were at the protonymph stage, because the previous experiment showed that more than 60% of the *P. macropilis* juveniles without food had died on the third day and, thus, we could no longer discriminate between death due to starvation or predation. Therefore, it was not possible to measure intraguild predation among the deutonymphs in this experiment. Moreover, we did not investigate each juvenile stage separately because their duration can vary and may not last a day.

### Statistics

Predation rates were compared with the Wilcoxon rank-sum test. To analyse the effects of feeding on heterospecific eggs on survival of juveniles, we used a time-to-event analysis (Cox proportional hazards model) using the function ‘coxph’ of the ‘survival’ package (R Development Core Team 2017). The Kaplan–Meier estimate, which takes censored data into account, was used to assess survival through time. Because none of the juveniles reached the deutonymph or adult stage in the controls, the proportions of juveniles reaching these stages were compared with a Pearson’s Chi-squared test. Oviposition rates of adult females were compared between treatments with a Wilcoxon rank-sum test. All analyses were performed with the statistical software R, version 3.3.3 (R Development Core Team 2017).

## Results

### IGP of adult females on heterospecific eggs, larvae and adult females

**Fig. 1:**
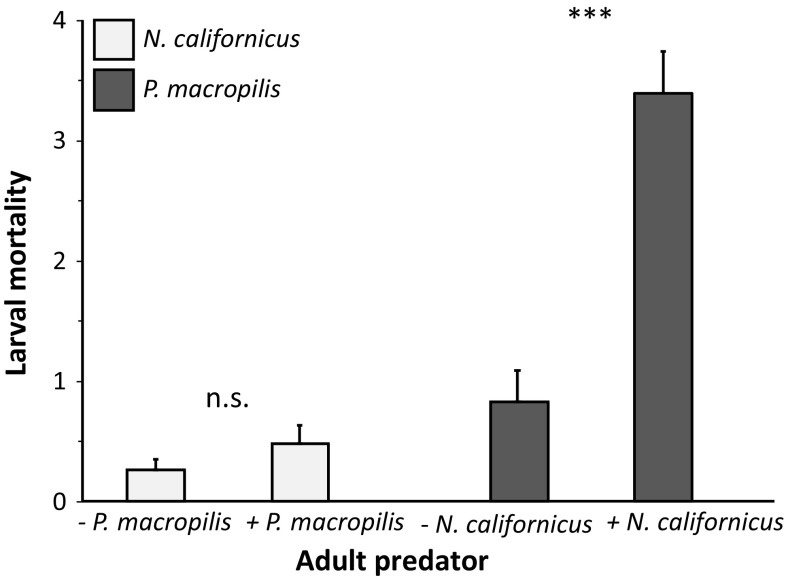
Average mortality (+ SE) of larvae of *N. californicus* (light bars) and *P. macropilis* (dark bars) after 24 h in the presence (+) or absence (−) of an adult female of the other species. Asterisks indicate significant effect of the presence of an adult predator on mortality within a larval species. ***: *P* < 0.001; *n.s* not significant

The mortality of eggs of *N. californicus* in the presence and absence of adult *P. macropilis* was zero. The mortality of *N. californicus* larvae in the presence of adult *P. macropilis* was low and not significantly different from mortality without adult *P. macropilis* (Fig. 1; Wilcoxon rank-sum test: *W* = 317, *P* = 0.35). We conclude that adults of *P. macropilis* are not intraguild predators of *N. californicus* eggs and larvae.

**Fig. 2:**
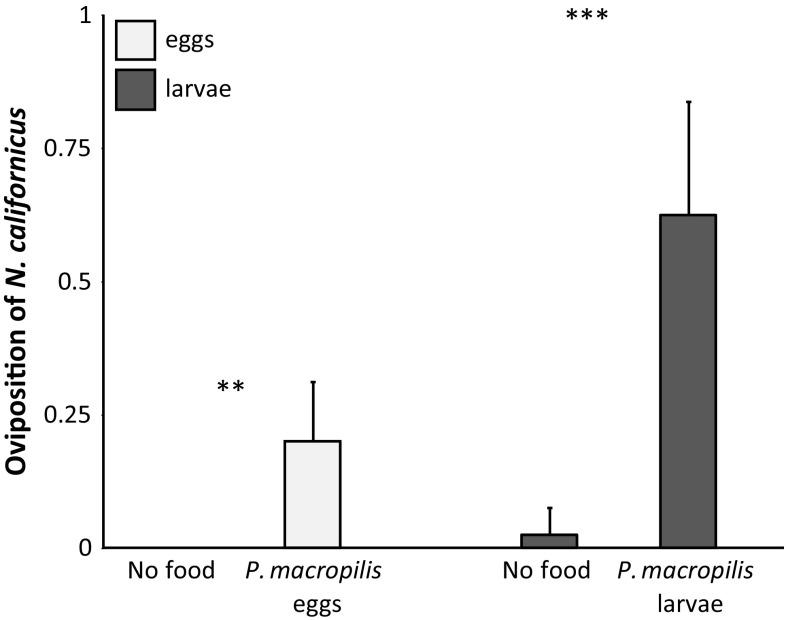
Average oviposition (± SE) of *N. californicus* fed on *P. macropilis* eggs (during 5 days, light bars) or larvae (during 4 days, dark bars) and without food (No food). Asterisks show significance of difference between the treatment without food and with intraguild prey. **: *P* < 0.01; ***: *P* < 0.001

In contrast, there was a significantly higher mortality of eggs of *P. macropilis* in the presence of adult females of *N. californicus* than in their absence (mortality with *N. californicus*: 1.14 ± 0.44 (SE) eggs, without *N. californicus*: 0.0 eggs; Wilcoxon rank-sum test: *W* = 140, *P* = 0.016). The mortality of larvae of *P. macropilis* was also higher in the presence of adult females of *N. californicus* than in their absence (Fig. 1; Wilcoxon rank-sum test: *W* = 294.5, *P* < 0.001). Adult females of *Neoseiulus californicus* did not oviposit in the absence of eggs of *P. macropilis*, and half of the females of *N. californicus* oviposited in the presence of eggs of *P. macropilis*. This difference was significant (Fig. 2; Wilcoxon rank-sum test: *W* = 147, *P* = 0.006). All adult females of *N. californicus* oviposited in the presence of larvae of *P. macropilis* and only one female produced one egg in their absence, and this difference was also significant (Fig. 2; Wilcoxon rank-sum test: *W* = 98, *P* < 0.001). We conclude that adult females of *N. californicus* are intraguild predators of *P. macropilis* eggs and larvae.

There was no mortality of adult females in the presence or absence of adult females of the other species.

### IGP of juveniles on heterospecific eggs and juveniles

**Fig. 3:**
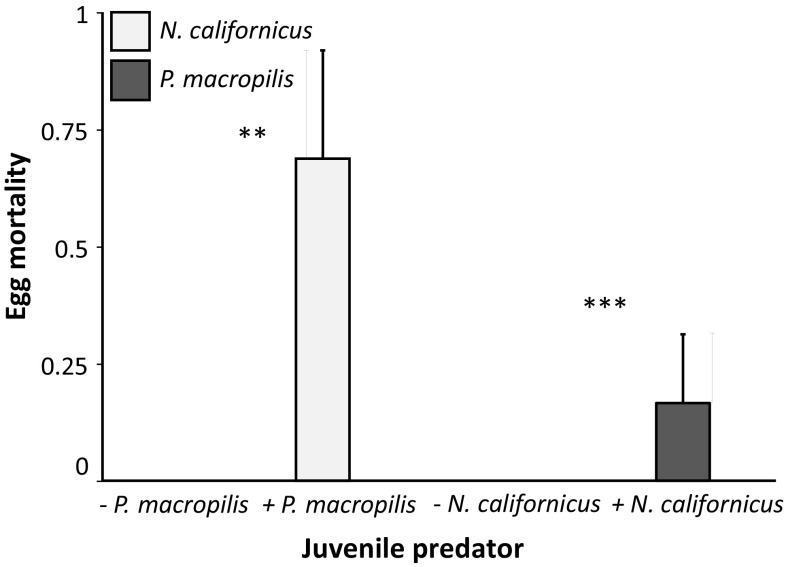
Average egg mortality (+ SE) of *N. californicus* (light bars) and *P. macropilis* (dark bars) in the presence (+) or absence (−) of juveniles of the other species. Asterisks indicate significant effect of the presence of a juvenile predator on mortality within eggs of a species. **: *P* < 0.01; ***: *P* < 0.001

**Fig. 4:**
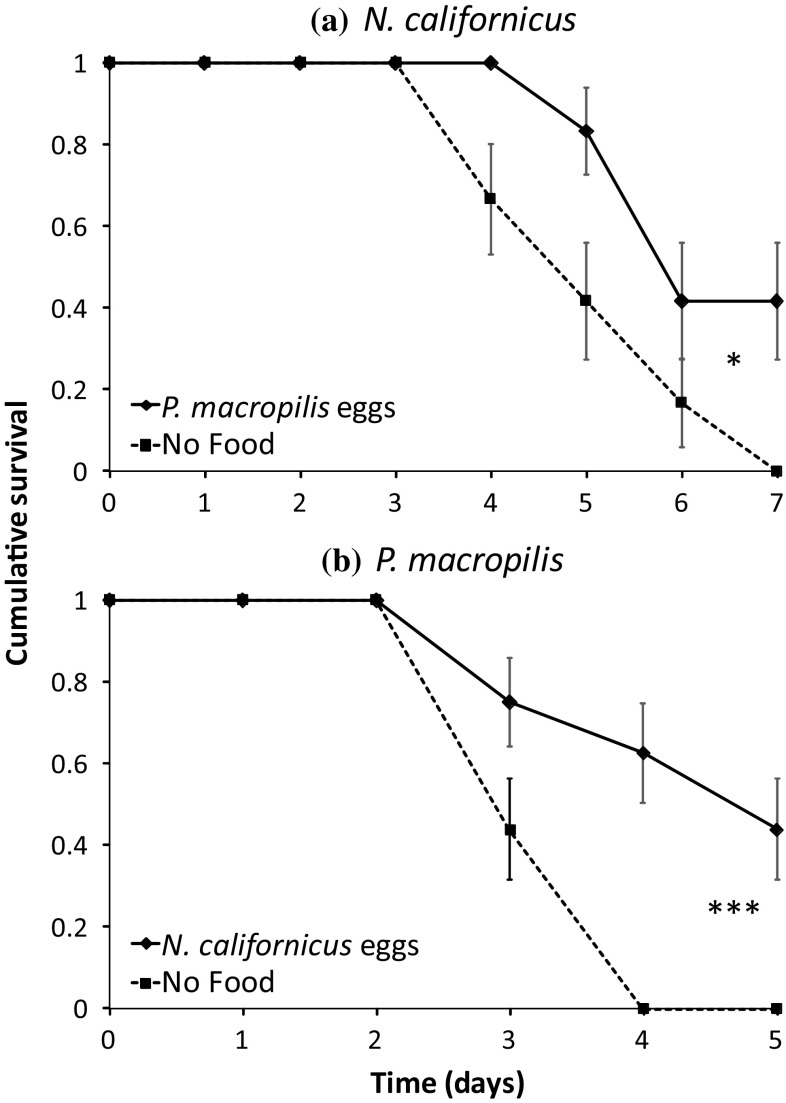
Cumulative survival (± SE) as a function of time of juveniles of *N. californicus* **a** and *P. macropilis*, **b** fed on heterospecific eggs (diamonds, solid line) and without food (No food, squares, dashed line). Asterisks indicate significance in survival between the two treatments per species. *: *P* < 0.05; ***: *P* < 0.001

Juveniles of both *N. californicus* and *P. macropilis* preyed on eggs of the other species until they reached adulthood (Fig. 3). This resulted in a significant mortality of the eggs (*P. macropilis* eggs: Wilcoxon rank-sum test: *W* = 120, *P* = 0.0013; *N. californicus* eggs: *W* = 232, *P* < 0.001). Juvenile survival of both species significantly increased by feeding on heterospecific eggs (*N. californicus*: Fig. 4a, Cox proportional hazards: log rank = 6.52, *df* = 1, *P* = 0.011; *P. macropilis*: Fig. 4b, Cox proportional hazards: log rank = 13.3, *df* = 1, P = 0.0003). In the absence of *P. macropilis* eggs, none of the *N. californicus* juveniles (*n* = 12) reached the deutonymph or adult stage, whereas 58% reached the deutonymph stage (Pearson *χ*^2^: 12.3, *df* = 3, *P* = 0.0063) and 33% reached adulthood (Pearson *χ*^2^: 13.3, *df* = 3, *P* = 0.0039) in their presence. Juveniles of *P. macropilis* (*n* = 16), did not develop into deutonymphs or adults in the absence of *N. californicus* eggs, but 63% reached the deutonymph stage (Pearson *χ*^2^: 17, *df* = 3, *P* = 0.0007) and 44% adulthood (Pearson *χ*^2^: 16.3, *df* = 3, *P* = 0.0010) in their presence. This shows that the juveniles kill heterospecific eggs and benefit from this, which are both prerequisites for IGP (Polis et al. 1989; Fonseca et al. 2017). We therefore conclude that juveniles of both species are intraguild predators of heterospecific eggs.

There was no mortality of larvae and protonymphs of the two species in the presence of the same stages of the other species (14 replicates). We conclude that juveniles (larvae and protonymphs) of both species are not intraguild predators of heterospecifics of the same stage.

## Discussion

**Fig. 5:**
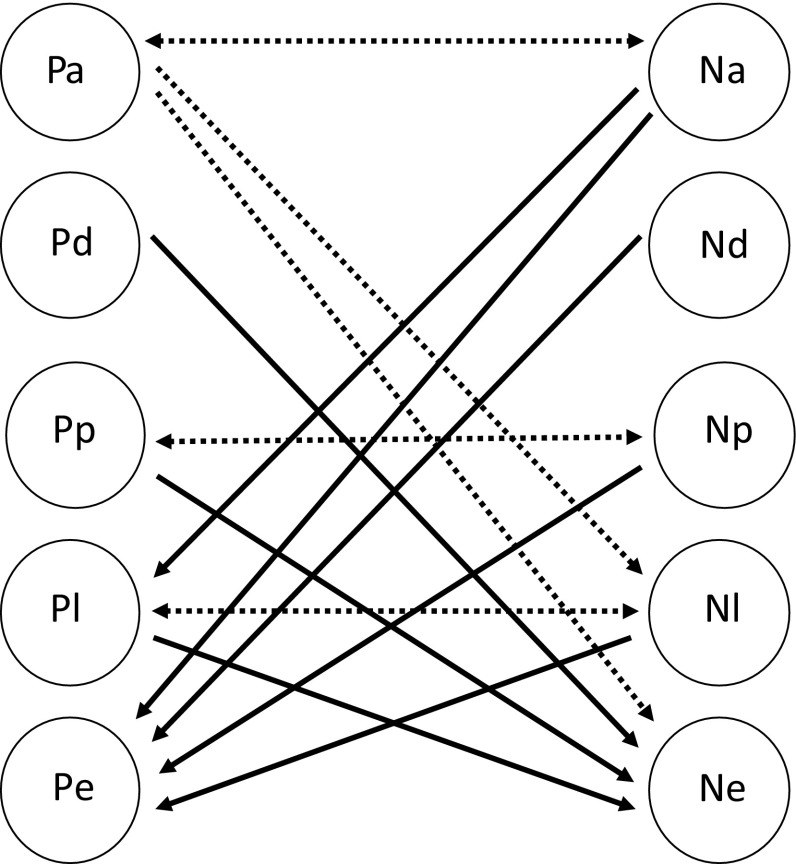
Intraguild predation among various stages of the predatory mites *P. macropilis* and *N. californicus*. The capital letters in the circles indicate the predator species (“P” for *P. macropilis* and “N” for *N. californicus*) and the normal type signifies the ontogenetic stages (“a” for adults, “d” for deutonymphs, “p” for protonymphs, “l” for larvae and “e” for eggs). Arrows point from attacker to victim. The solid lines indicate the occurrence of intraguild predation and dashed lines indicate its absence

Our results show that ontogeny clearly plays a critical role in determining the occurrence of intraguild predation (IGP) within our predator system. Figure 5 summarizes the results and shows the complex interactions occurring between these two species. So far, most studies on IGP in arthropods have focused on adult individuals preying on juveniles (but see Guo et al. 2016). In contrast, we show here that juvenile stages can be IG predators but adults are not: whereas the juveniles of *P. macropilis* develop from larva until adult when feeding on *N. californicus* eggs, the large adult females of *P. macropilis* did not feed on the smaller stages (eggs and larvae, Fig. 5) of *N. californicus*. It is therefore crucial to investigate IGP by all ontogenetic stages of the species involved. Because IGP is frequently associated with complex life cycles (Polis et al. 1989) and because ontogenetic shifts represent the mode of life in 80% of animal taxa (Werner 1988), similar phenomena are likely to occur in many other IGP systems. Moreover, this demonstrates the complexity of interactions among stage-structured populations (de Roos and Persson 2013) and the ways in which stage-structured IG predators and IG prey can interact. We furthermore show that IGP in our system was reciprocal (Fig. 5). Although there was no IG predation in the experiments between juveniles of the same stages of both species, and also not between adult females, both juveniles and adult females of *N. californicus* were IG predators of smaller stages of *P. macropilis*.

Besides stage structure, another criterion for assessing the possible occurrence of IGP is that the predation should result in direct nutritional and energetic gains, i.e. increased growth, reproduction or survival (Polis et al. 1989; Fonseca et al. 2017). Here, we show that juveniles of *P. macropilis*, a species considered as a highly specialized predator of Tetranychid mites (McMurtry and Croft 1997), can complete their juvenile development feeding only on IG prey. *Neoseiulus californicus* was classified as a selective predator of Tetranychids (McMurtry and Croft 1997; Croft et al. 1998), but its juveniles also develop until adulthood feeding only on IG prey, and 50 and 100% of the adult females reproduced on eggs or larvae of *P. macropilis*, respectively. We therefore suggest that the concept of specialist predatory mites used in the literature may be too strict (McMurtry and Croft 1997). Because many predator–prey systems in nature are ephemeral, organisms will commonly encounter situations without prey and therefore have to adapt to feed on other available resources. Thus, what defines a predator’s diet, are the encounter rates with different prey types (Rosenheim et al. 2004) and its ontogenetic stage. In case of IGP, such adaptations to alternative food possibly originate from interference competition because there will probably be selection for those predators that do not only kill their competitors, but also feed on them (Fonseca et al. 2017). Subsequently, selection can act on the IG predators to be more efficient at converting IG prey (Polis 1988; Polis et al. 1989; Fonseca et al. 2017).

Considering a three-species module (an IG predator, an IG prey and a shared prey), IGP may in theory result in the exclusion of one of the predator species (Diehl and Feissel 2000, 2001). For coexistence of all three species, the IG prey must be superior at exploitative competition for the shared resource, and even then coexistence is only possible at intermediate levels of productivity (Polis and Holt 1992; Holt and Polis 1997; Mylius et al. 2001). Nevertheless, IGP is thought to be ubiquitous in nature (Polis et al. 1989; Arim and Marquet 2004), and several ecological factors such as structural complexity of the habitat (Finke and Denno 2002; Warfe and Barmuta 2004; Harvey and Eubanks 2005; Griffen and Byers 2006; Janssen et al. 2007; but see Reichstein et al. 2013) and the presence of alternative resources (Holt and Huxel 2007; Daugherty et al. 2007) can increase the possibilities for coexistence. Several theoretical and empirical studies have shown that coexistence of IGP predators and prey may sometimes be enhanced by stage structure (Mylius et al. 2001; Borer 2002; Hin et al. 2011; Schellekens and van Kooten 2012). However, other experimental and theoretical studies demonstrated that the stage structure of species involved in IGP reduced the scope of coexistence (van de Wolfshaar et al. 2006; Persson et al. 2007b; Montserrat et al. 2008, 2012; Schröder et al. 2009; Reichstein et al. 2013; Toscano et al. 2016). In conclusion, it is clear that stage structure does affect the dynamics and persistence of populations involved in intraguild predation, not in the least because it can lead to reciprocal IGP. We therefore suggest that models of IGP could include more complex stage structure, and empirical studies should consider the interactions among all stages of interacting species.

There is growing awareness that ontogenetic shifts among trophic levels may have profound effects on the structure and dynamics of food webs because they result in size- or age-structured interactions (Polis 1984; Cohen et al. 1993a, b; Woodward et al. 2005; Rudolf 2006, 2007; Rudolf and Lafferty 2011; de Roos and Persson 2013). Yet, community matrices often represent networks of feeding links among species (Pimm et al. 1991; De Ruiter et al. 2005; Jonsson et al. 2005), but not among stages of species, and these stages may have different ecological roles with respect to a particular other species. Because the large majority of animal populations are stage, age, or size structured (Werner 1988; de Roos and Persson 2013), we suggest that feeding links in community matrices should be represented at levels lower than the species (see Preston et al. 2012, 2014). Although scientists strive for generalizations and simplifications, and with good reasons, we should always be aware of the intrinsic complexity of natural ecosystems and interactions (Botkin 1990; Meyer 1993). If we would have studied intraguild predation of only adults on juveniles instead of the interactions among various stages of the two predators, we would have concluded that the two species were involved in simple intraguild predation instead of reciprocal IGP. Thus, our results show that interactions among all stages of species with complex life cycles should be studied to increase our understanding of community dynamics.
